# Neuronal mechanisms underlying binaural performance with cochlear implant in single-sided deafness: a [^15^O]water PET study

**DOI:** 10.1007/s00259-025-07639-8

**Published:** 2025-11-15

**Authors:** Iva Speck, Susan Arndt, Johannes Thurow, Lars Frings, Thomas Wesarg, Antje Aschendorff, Philipp T. Meyer, Ganna Blazhenets

**Affiliations:** 1 Department of Nuclear Medicine, Medical Center - University of Freiburg, Hugstetterstr. 55, Freiburg im Breisgau, 79106 Germany; 2 Department of Otorhinolaryngology - Head and Neck Surgery, Medical Center - University Freiburg, Freiburg, 79106 Germany

**Keywords:** Unilateral deafness, Single-sided deafness, Cochlear implant, CI, Water-PET

## Abstract

**Purpose:**

We examined neuronal activation patterns in single-sided deaf (SSD) cochlear implant (CI) recipients and normal-hearing (NH) controls to unravel neuronal mechanisms underlying binaural integration in SSD-CI.

**Methods:**

20 SSD-CI (CI left) and 10 NH controls underwent [^15^O]water PET (assessing relative cerebral blood flow, rCBF) during auditory stimulation (forward or time-reversed, unilaterally to NH ear or bilaterally). Subjects were divided into SSD-CI with good (≥ 80%) and poor (< 80%) performance based on a median split of correct word understanding in PET background noise. Voxel-wise analyses identified volumes of interest for regional analyses (right primary auditory cortex (PAC), left superior temporal gyrus (STG)) for volume-of-interest analyses.

**Results:**

Compared to SSD-CI with good performance, those with poor performance showed poorer word understanding during unilateral stimulation of the NH ear. The activation of the right PAC was significant and comparable across groups. The asymmetry index of rCBF of the PAC decreased significantly in NH controls and SSD-CI with good performance from uni- to bilateral stimulation, but not in SSD-CI with poor performance. A significant activation of left STG was only observed in NH controls and SSD-CI with good performance.

**Conclusion:**

This study underlines the usefulness of [^15^O]water PET to explore regional neuronal mechanisms underlying CI performance: SSD-CI with poor performance are characterized by a low normal word understanding with the NH ear only (in line with the lack of STG activation) and compromised binaural integration, although the CI provides quantitatively comparable input to the contralateral PAC in both SSD-CI groups.

**Supplementary Information:**

The online version contains supplementary material available at 10.1007/s00259-025-07639-8.

## Introduction

The World Health Organization (WHO) estimates that about 5% of the world’s population has impaired hearing, with hearing loss being the third leading cause of years lived with disability [1]. The Lancet Commission on dementia prevention, intervention, and care estimated that hearing loss in midlife constitutes the most important single modifiable risk factor for dementia, accounting for a population-attributable fraction of 7% (45% in total for all 14 risk factors) [2]. Single-sided deafness (SSD) is a commonly neglected form of hearing loss that is defined as profound hearing loss in one ear with normal or nearly normal hearing preserved in the other ear [3], which affects about 7.9% to 13.3% of the world’s population [4]. In subjects with SSD, the loss of binaural integration (i.e., the ability to combine auditory information from both ears) impairs speech understanding in background noise and also localization of sound sources [5, 6], which often leads to exhaustion, frustration, difficulties in interpersonal communication, and may cause social isolation. SSD can additionally cause social isolation as a result of heightened stress levels and increased listening effort [6–9]. Many of those affected also experience tinnitus, which they often perceive as disturbing [10–12].

The only treatment to at least partially restore binaural hearing in subjects with SSD is a cochlear implant (CI). CI is a neural device that enables speech recognition through electrical stimulation of the auditory nerve. Treatment with CI in subjects with SSD (partly) restores binaural integration, improves speech recognition in quiet and noise, localization of sound sources, tinnitus control, and overall quality of life [10, 13–16]. Treatment with CI is superior to therapy with contralateral routing of signal hearing aid, bone-anchored hearing aid, or no therapy [17–19]. Yet, the outcome after CI treatment in SSD is highly variable. To date, there is only a limited understanding of neural changes after CI treatment that might explain and help to predict variability in outcome. Adults with SSD showed a significant positive correlation with residual hearing in the implanted ear [20] and a significant negative association between speech recognition in noise with CI and duration of deafness [20, 21]. However, duration of deafness had no significant impact on CI usage 12 and 24 months after implantation in subjects with SSD [22].

Neuronal mechanisms underlying treatment success with CI are unclear, and a better understanding could help identify patients who will benefit most from CI implantation. This knowledge can reduce associated side effects and risks of CI treatment with suboptimal outcomes and minimize costs. To identify these mechanisms, the present study explores neuronal processes involved in word understanding in noise – one key difficulty for subjects with SSD – employing positron emission tomography (PET) in combination with the freely diffusible radiotracer [O]water. [^15^O]water PET allows us to image the regional cerebral blood flow (CBF) as a marker of regional neuronal activity and examine task-dependent changes. PET offers the advantage that it can be safely used in subjects with CI, in contrast to functional magnetic resonance tomography. In addition, PET is a quieter imaging technique (ambient noise around 77 dB [23]), which interferes less with acoustic stimuli.

We aimed to identify the association between neuronal activity patterns in SSD-CI subjects and binaural word understanding in noise with CI. Based on our prior research [24, 25], we hypothesized that SSD-CI subjects with good and poor performance would show differences in activation of the primary auditory cortex (PAC), a region involved in speech perception. We further hypothesize that SSD-CI subjects and normal-hearing (NH) controls differently activate the left superior temporal gyrus (STG, comprising “Wernicke’s area”), a region responsible for speech understanding.

## Materials and methods

### Subjects

20 SSD-CI subjects and 10 NH controls were prospectively recruited. Subjects with CI were recruited if they (1) were right-handed (confirmed by the Edinburgh handedness inventory [26]), (2) treated with CI on the left side, (3) had at least six months of CI experience, and (4) aged at least 18 years. Left-sided SSD was defined according to the consensus paper by Vincent et al. [3]. As the NH control group, we recruited (1) right-handed subjects, (2) aged at least 18 years, with (3) verified normal hearing. Normal hearing was defined as a mean air-conduction hearing threshold of 20 dB HL or below for the frequencies 500, 1000, 2000, and 4000 Hz. Exclusion criteria for both groups were: (1) relevant neurological and/or psychiatric disease (e.g., cognitive impairment, epilepsy, cerebrovascular disease, brain tumor, depression) or (2) pregnancy.

### Examination and hearing measurements

Each subject underwent a microscopic otoscopy with the removal of cerumen if present. SSD-CI subjects also underwent clinical examination of the implantation scar and the local implant site. For each of the SSD-CI subjects, residual hearing (pure-tone hearing threshold of the poorer-hearing ear before treatment with CI), duration of deafness, CI experience, and age at onset of deafness were also collected.

### CI adjustment

All SSD-CI subjects were equipped with a loaner sound processor to minimize the possible impact of differing preprocessing of sounds. All SSD-CI subjects who used a Nucleus implant (Cochlear Ltd., Lane Cove, Australia) were equipped with either the CP910 (CI24RE CA, CI512, CI522, or CI532) or the CP1000 (CI612 or CI632). Subject SSD5, who used the SYNCHRONY FLEX 24 (MED-EL GmbH, Innsbruck, Austria), was provided with a loaner sound processor SONNET. Each subject’s favorite everyday program setting was transferred to the loaner processor.

To ensure comparable subjective loudness of the NH ear and CI, a burst of white noise at 65 dB SPL was sequentially presented to the NH ear via headphone and to the CI sound processor via wireless streaming or direct audio input. We adjusted the voltage of the direct audio input signal until the SSD-CI subject reported equal loudness with the NH ear and CI.

### Word understanding in noise

We applied the Oldenburg sentence test (OLSA) [27, 28] to examine word understanding in PET background noise and additionally as an auditory stimulus during the PET scans. Based on this measure, SSD-CI subjects were split into SSD-CI with good or poor performance, with the cutoff defined as the median word understanding in the PET background noise of the whole SSD-CI group. Subjects were asked to repeat each word in 30 5-word sentences from a randomly selected list and guess words if unsure. The sentences are grammatically correct but semantically unpredictable and void of meaning. This ensures that the OLSA results reflect the word understanding and not partially the subject’s ability to guess.

Word understanding in noise was assessed after the PET measurement in the scanner room. During the hearing measurement, the subject lay on the bed of the PET/CT scanner at the position in which the subject underwent the PET scans (i.e., in comparable PET noise).

The Vereos PET/CT scanner (Philips Healthcare, The Netherlands) produces background noise at a mean level of 76.9 dB SPL. Background noise and its effect on word understanding in PET background noise was previously described for NH subjects [23].

The sentences were presented via an IP30 insert phone (Radioear, Denmark) to the NH ear. We placed the insert phone without an E-A-RLINK 3B foam ear tip (3M company auditory systems, USA) in the auditory canal to leave the auditory canal open. We presented the auditory stimuli directly via audio cable attached to the direct audio input jack of the CP910 and SONNET or wirelessly to the CP1000, which is not equipped with a direct audio input, using the MiniMic 2+ (Cochlear Ltd., Australia). We have chosen a mixing ratio of 1:1 between direct audio input/wireless input (speech stimulus) and microphone of the sound processor (PET noise). PET noise is produced by the PET scanner at a level of 76.9 dB SPL, while OLSA sentences are presented at a level of 72.9 dB SPL, resulting in a signal-to-noise ratio (SNR) of −4 dB. We chose this particular SNR as it resulted in a speech recognition score of 50% during unilateral stimulation in NH controls. A challenging auditory situation in which NH controls understand only 50% correctly is most suitable to detect benefits from bilateral stimulation.

We measured the speech recognition score unilaterally (only right NH ear) and bilaterally (NH ear and CI or both NH ears). To achieve unilateral stimulation in NH controls, we masked the left ear using an earplug OHROPAX^®^ Soft (Wehrheim, Germany) and a circumaural headphone (3M™ PELTOR™ X5, Germany). In SSD-CI subjects, the CI was turned off.

Before testing word understanding, each subject underwent training in a soundproof test room in quiet and in recorded PET noise the day before PET measurement (details see [23]).

## PET scans

### Stimuli

During PET scans, we stimulated the subjects with two different auditory stimuli: During sentence presentation, we presented a list of 30 randomly selected OLSA sentences with a 0.5-second pause between each sentence. In the time-reversed (TR) sentence presentation, we presented TR OLSA sentences that were unintelligible but had similar acoustic properties to usual OLSA sentences. Both acoustic stimuli were presented unilaterally to the right NH ear and bilaterally to the NH ear and CI or both NH ears. No stimulation of the CI alone was performed.

### Design

**Fig. 1:**
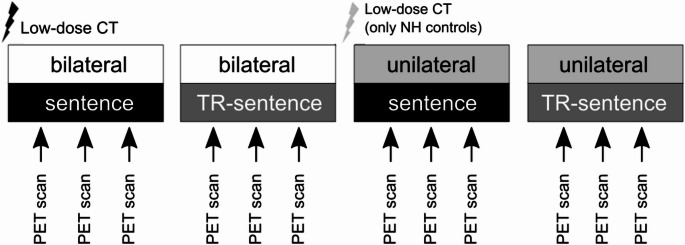
Diagram of exemplary PET protocol. NH controls received an additional low-dose CT, as placing/removing of headphones and earplugs resulted in head movement. NH, normal-hearing; TR-sentence, time-reversed sentence

The experimental design included three factors: (1) a between-subjects factor ‘group’ with three conditions (NH controls vs. SSD-CI subjects with good performance vs. SSD-CI subjects with poor performance); (2) a within-subjects factor ‘side of stimulation’ with two conditions (unilateral vs. bilateral); and (3) a within-subjects factor ‘kind of stimulation’ (with two conditions: sentence vs. TR-sentence). This resulted in 4 conditions for each group: (A) bilateral/sentence, (B) bilateral/TR-sentence, (C) unilateral/sentence, and (D) unilateral/TR-sentence (Fig. 1). The sequence of test conditions was randomized across subjects in a block design. For the analysis, the following contrasts were formed: (1) bilateral > unilateral stimulation, termed ‘side of stimulation’; and (2) sentence > TR-sentence presentation, termed ‘kind of stimulation’.

### PET acquisition and image processing

CBF as a surrogate marker of neuronal activity was measured with [^15^O]water PET. All subjects underwent PET measurements on a fully digital Vereos PET/CT scanner. Three scans for each of the conditions were acquired for each subject, resulting in 12 PET scans per subject. For each PET scan, 295 ± 3 MBq [^15^O]water was administered intravenously as a bolus injection with the subject being positioned in the field of view of the scanner. 4-minute list mode scans were started with the injection of the tracer. Using low-dose CT for attenuation correction, 15 to 75 seconds post injection static datasets (i.e., 1-min scan after tracer arrival in the brain, reflecting CBF) were reconstructed with the vendor-specific, line-of-response time-of-flight ordered-subsets 3-dimensional iterative reconstruction algorithm using spherically symmetric basis functions (so-called blob ordered-subset time-of-flight reconstruction; number of iterations, 5; number of subsets, 11; 2 mm Gaussian post filtering; point spread function recovery off; resulting voxel size, 1.0 × 1.0 × 1.0 mm).

### Image analysis

All processing steps were implemented with an in-house pipeline in MATLAB (The MathWorks, Inc.) and Statistical Parametric Mapping (SPM) 12 software (www.fil.ion.ac.uk/spm). PET scans were spatially normalized to an in-house PET template of comparable spatial resolution in MNI space and scaled to the mean counts in brain parenchyma for assessment of the relative CBF (rCBF).

To verify the appropriateness of the selected conditions and to define volumes of interest (VOI) for subsequent group-wise regional analyses, we performed full-brain voxel-wise SPM analyses for comparison between stimuli across all subjects using a full-factorial design (general linear model framework), accounting for repeated measures and adjusting for subjects’ age. For this analysis, rCBF maps of all subjects were smoothed with an isotropic Gaussian kernel of 10 mm full width at half maximum to average small anatomical differences. Contrasts ‘side of stimulation’ (bilateral vs. unilateral) and ‘kind of stimulation’ (sentence vs. TR-sentence) were explored. The following contrasts were constructed: bilateral > unilateral stimulations to reveal blood flow response associated with stimulation of the left (CI) ear, and sentence > TR-sentence presentations to reveal blood flow response associated with word understanding. Voxels with family-wise error (FWE)-corrected *p* < 0.05 at peak-level (cluster extent of at least 100 voxels corresponding to 0.1 mL) were considered statistically significant. Identified unique clusters were converted to VOI and were used as cohort-specific masks to extract rCBF for further regional analyses. Mean rCBF values and stimulation-dependent differences were compared between NH controls and SSD-CI subjects.

### Statistical analysis

Statistical analyses were performed with the R software (version 4.1.0, http://www.R-project.org). Differences in demographic, clinical, and auditory test variables were assessed by ANOVA or Chi-squared test. Significant differences in ANOVA were followed by Wilcoxon rank tests with Bonferroni adjustment. Differences in mean regional rCBF between conditions were tested with repeated-measures ANCOVA adjusted for age. ANCOVA was followed by pairwise least-squares means comparison with Tukey multiplicity adjustment. Task-specific activation (Δ) was calculated as the percent difference between mean regional rCBF for each condition.

Additionally, we investigated the effects of the lateralization of neuronal activations in response to stimulation. These effects were assessed by the asymmetry index (AI) between the left (ipsilateral to CI in SSD-CI subjects) PAC and right PAC (contralateral to CI in SSD-CI subjects). We used individual differences in regional rCBF of the atlas-defined PAC adopted from Morosan et al. [29] (see Speck et al. [24] for details).

1 $$\:AI-PAC\:\left[\%\right]=\:\frac{{rCBF}_{left}-{rCBF}_{right}}{{rCBF}_{left}+{rCBF}_{right}}\cdot\:200$$

We calculated the AI-PAC for each ‘side of stimulation’ condition (i.e., uni- and bilateral stimulation), combining sentences and TR-sentences. We compared AI-PAC values during unilateral stimulation with bilateral stimulation using a paired Wilcoxon rank test, pairing AI-PAC by subject and ’kind of stimulation’.

For all comparisons, *p* < 0.05 (adjusted for multiple comparisons) was considered statistically significant.

## Results

### Study populations

20 SSD-CI subjects and 10 NH controls were recruited into this prospective study. One NH control was excluded due to technical problems with the provided stimulation. All subjects reported normal eyesight.

**Fig. 2:**
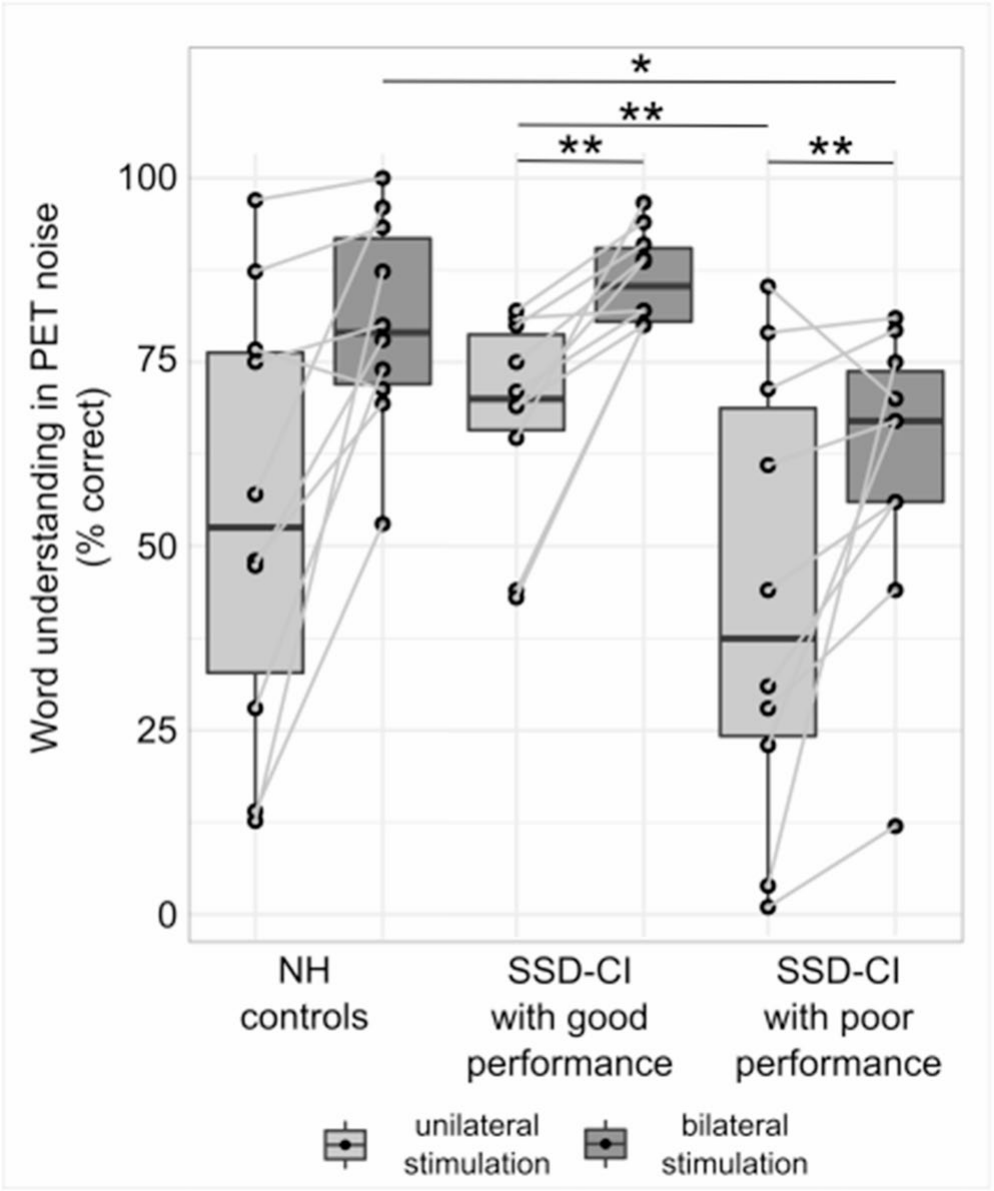
Word understanding in PET noise at an SNR of −4 dB (expressed as % correct for NH controls, SSD-CI subjects with good and poor performance). *p < 0.05; **p < 0.01. Of note, word understanding in noise with bilateral stimulation was not statistically compared between SSD-CI subjects with good and poor performance because this parameter was used for group definition. CI, cochlear implant; NH, normal-hearing; SSD, single-sided deafness

To evaluate the performance during bilateral simulation, we used a median split of correct word understanding in PET background noise during bilateral stimulation (median = 80%). This split resulted in two groups: SSD-CI subjects with good performance (≥ 80% recognition during bilateral stimulation, *n* = 10) and SSD-CI subjects with poor performance (< 80% recognition during bilateral stimulation, *n* = 10), (Fig. 2). The SSD-CI subjects with good and poor performance did not differ in deafness duration, age at onset of deafness, and CI experience (Table 1).

**Table 1:** Characteristics of included subjects with SSD and NH controls

Parameter	SSD-CI subjects (all)	SSD-CI subjects with *good* performance		SSD-CI subjects with *poor* performance	NH controls	ANOVA or Chi-squared
n	20	10		10	9	
age	47 ± 12 [20; 65]	43 ± 14** [21; 65]		48 ± 11** [30; 64]	30 ± 10** [21; 52]	*p* < 0.01
sex	F, *n* = 10 M, *n* = 10	F, *n* = 5 M, *n* = 5		F, *n* = 5 M, *n* = 5	F, *n* = 5 M, *n* = 4	n.s.
Air-conduction 4PTA [dB HL]/Aided threshold 4PTA [dB HL]						
right NH ear	10.2 ± 4.7 [2.5; 20]	9.1 ± 5.0 [2.5; 20.0]		11.3 ± 4.3 [6.3; 18.8]	9.7 ± 4.0 [3.8; 16.39]	n.s.
left NH ear					11.1 ± 5.73 [2.5; 18.8]	N/A
Left hearing-impaired ear°	105 ± 24.7 [70; 130]	105.2 ± 23.5 [70; 130]	105.2 ± 24.7 [70; 130]			N/A
left CI ear	36.0 ± 6.4 [26.3; 50.0]	32.5 ± 3.8^#^ [26.3; 38.8]		39.5 ± 6.6^#^ [26.3; 50.0]	N/A	N/A
OLSA word recognition in PET noise with − 4 dB SNR [% correct]						
unilateral (right NH ear)	55.2 ± 27.5 [1.0; 85.3]	68.9 ± 15.4^#^ [43.0; 82.0]		41.5 ± 30.9^#^ [1.0; 85.0]	49.6 ± 27.3 [12.7; 87.0]	*p* < 0.001
bilateral	73.5 ± 19.8 [12; 96.7]	86.4 ± 6.2^##^ [81.0; 96.7]		60.6 ± 20.5^##^,* [12.0; 79.3]	78.0 ± 13.3* [53.0; 96]	
Clinical parameters						
Age at onset of deafness [years]	39 ± 12 [17; 59]	41 ± 13 [18; 59]		37 ± 12 [17; 52]	N/A	N/A
Duration of deafness [years]	4 ± 6 [0.1; 23]	2 ± 3 [0.2; 9]		6 ± 9 [0.4; 23]	N/A	N/A
CI experience	4 ± 3 [1; 11]	3 ± 2 [1; 7]		5 ± 4 [1; 11]	N/A	N/A

Mean ± standard deviation [range]; *p < 0.05, **p < 0.01 and ***p < 0.001 for comparison vs. NH controls and ^#^*p *< 0.05, ^##^*p *< 0.01 and ^###^*p *< 0.001 for comparisons between SSD-CI subgroups (t-test with Tukey multiplicity adjustment), °residual hearing before treatment with CI; *CI* cochlea implant, *dB* decibel, *NH* normal-hearing, *OLSA* Oldenburg sentence test, *4PTA* pure-tone average of 0.5, 1, 2 and 4 kHz, *SNR* signal-to-noise ratio, *SSD* single-sided deafness

During bilateral stimulation, word understanding in PET background noise was significantly lower in SSD-CI subjects with poor performance compared to NH controls (*p* < 0.05) and, by definition, compared to SSD-CI subjects with good performance. Likewise, word understanding during unilateral stimulation of the NH ear was significantly worse in SSD-CI subjects with poor compared to good performance (*p* < 0.01), but both SSD-CI subject groups did not differ from NH controls (Table 1). SSD-CI subjects with good performance showed high normal word understanding during unilateral stimulation and SSD-CI subjects with poor performance had low normal word understanding during unilateral stimulation (NH ear only).

The pure-tone hearing threshold (air conduction 4PTA) did not differ between the NH right ear in NH controls and the NH right ear in SSD-CI subjects (Table 1).

### Across group activation patterns

**Fig. 3:**
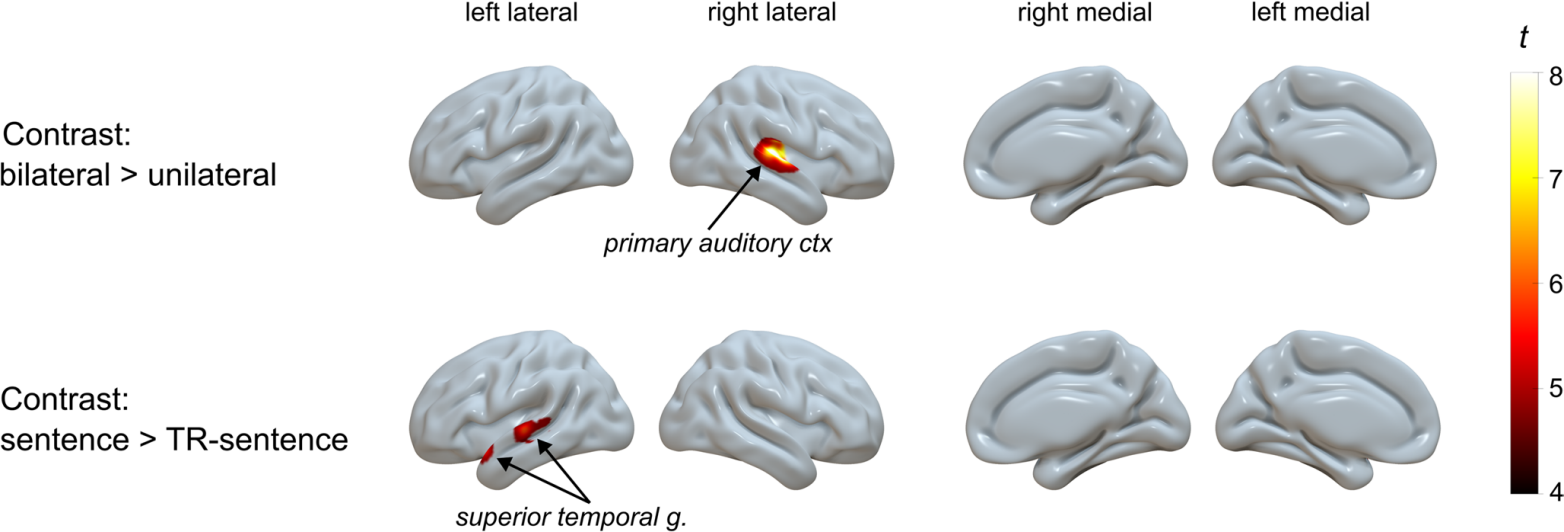
Blood flow response patterns for different stimulation conditions as assessed by voxel-wise full factorial analysis adjusted for individuals’ age. Statistical maps are thresholded at FWE-corrected p-value of 0.05. Activation patterns in each group separately are shown in Supplemental Fig. 1

Whole-brain voxel-wise activation patterns across pooled groups for the different stimulation conditions showed a significant effect of the contrast ‘side of stimulation’ in the right PAC (position of the peak: 45, −21, 6 mm) (contralateral to the CI in SSD-CI subjects) with stronger increase of rCBF at bilateral compared to unilateral stimulation (Fig. 3). When exploring the contrast ‘kind of stimulation’, we found a significant difference in the left STG (position of the peak: −52, 11, −13 mm) with rCBF being increased during sentence compared to TR-sentence presentation. These results confirm our expectation that the addition of left ear stimulation compared to unilateral right ear leads to a higher activation of the right PAC, while stimulation with sentence compared to TR-sentence content leads to a stronger activation of the left STG. These unique clusters were converted to VOI for further regional analyses.

### Group-related regional rCBF differences

#### Auditory input (‘side of stimulation’)

**Fig. 4:**
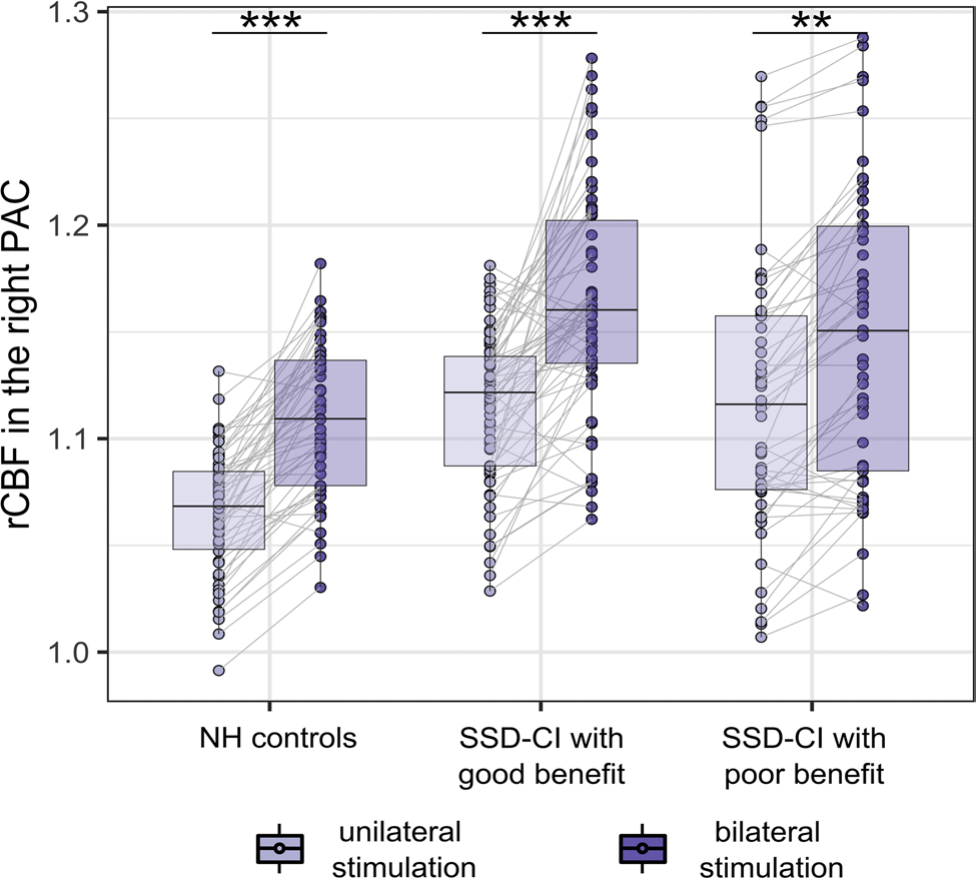
Regional rCBF during bilateral versus unilateral stimulation in NH controls and SSD-CI subjects with good and poor performance. Significance of difference (activation): **p < 0.01; ***p < 0.001. CI, cochlear implant; NH, normalheari-ng; PAC, primary auditory cortex; rCBF, relative cerebral blood flow; SSD-CI, single-sided deaf CI subjects

To assess the impact of ‘side of stimulation’ on the right PAC, we pooled sentence and TR-sentence presentation conditions. NH controls (Δ = 4.1% ± 1.5%, *t*(310) = 5.0, *p* < 0.001), as well as SSD-CI subjects with good performance (Δ = 4.2% ± 3.3%, *t*(310) = 6.0, *p* = 0.001) and poor performance (Δ = 2.7% ± 1.4, *t*(310) = 3.5, *p* = 0.008) exhibited significantly increased rCBF of the right PAC during bilateral stimulation (Fig. 4). No significant differences in activation magnitudes between NH controls and both SSD-CI groups were found, neither during the bilateral nor during the unilateral condition (*p* > 0.1). We found no effects of the repetition (order of the stimulations within the subject) on the rCBF in the right PAC (*p* > 0.9).

**Fig. 5:**
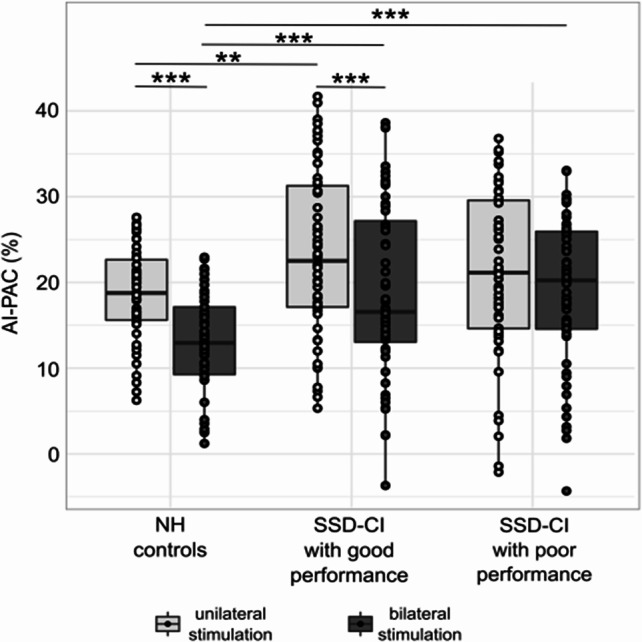
Asymmetry index of regional rCBF in the primary auditory cortex during bilateral versus unilateral stimulation in NH controls and SSD-CI subjects with good and poor performance. Significance of difference: **p < 0.01; ***p < 0.001. CI, cochlear implant; NH, normal-hearing; PAC, primary auditory cortex; rCBF, relative cerebral blood flow; SSD-CI, single-sided deaf CI subjects

NH controls (*p* < 0.001) and SSD-CI subjects with good performance (*p* < 0.001) showed significantly lower AI-PAC of regional rCBF during bilateral compared to unilateral stimulation (difference in AI-PAC: NH controls, −5.9% ± 4.1%; SSD-CI subjects with good performance, −4.3% ± 10.0%; Fig. 5). In contrast, SSD-CI subjects with poor performance showed no significant difference in AI-PAC of regional rCBF between uni- and bilateral stimulation (−1.7% ± 6.8%, *p* > 0.1). NH controls showed lower AI-PAC of the regional rCBF than SSD-CI subjects with good performance during uni- and bilateral stimulation (*p* < 0.01) and SSD-CI subjects with poor performance during bilateral stimulation (*p* < 0.001).

### Sentence processing (‘kind of stimulation’)

**Fig. 6:**
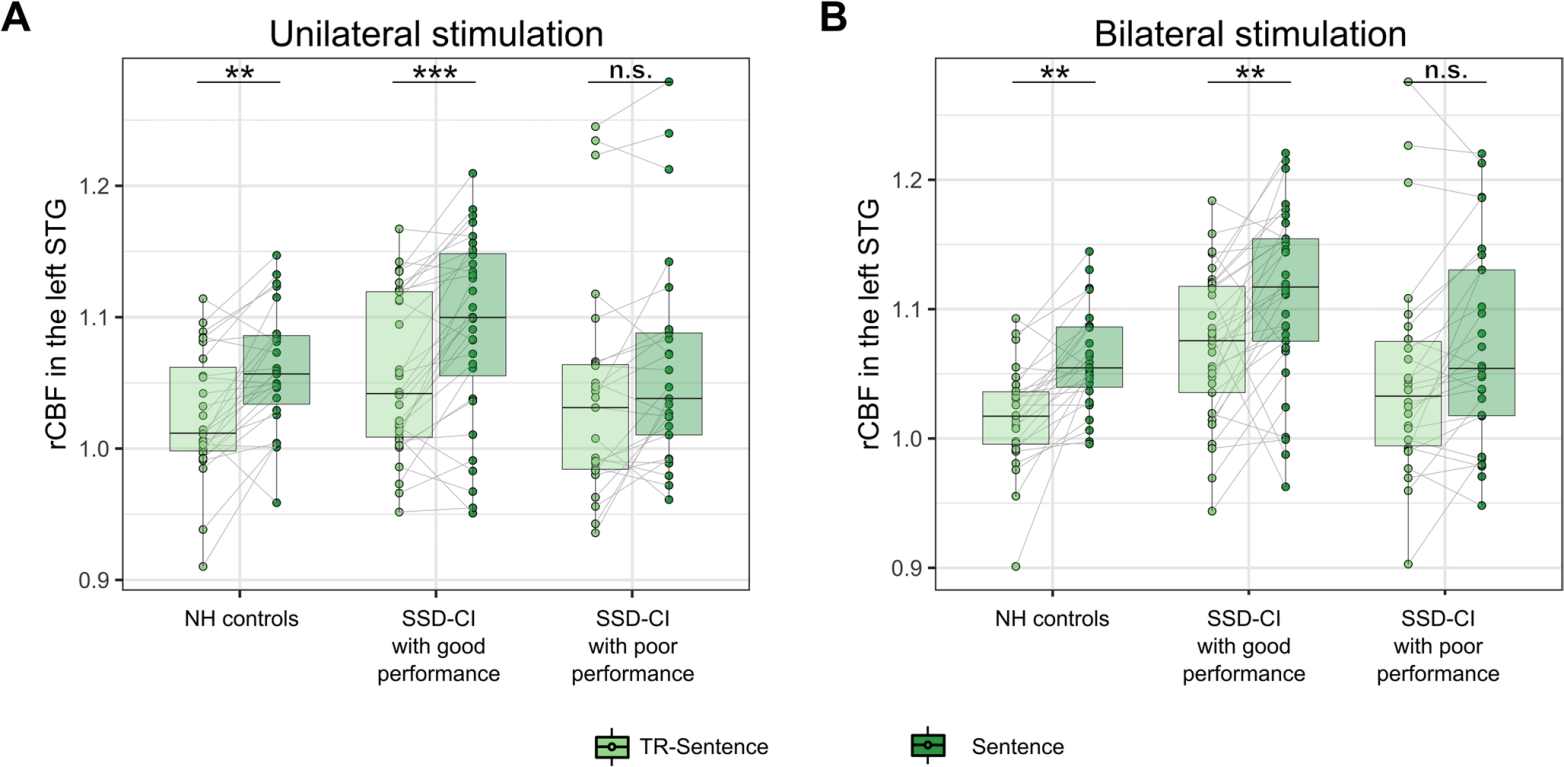
Regional rCBF during sentence vs. TR-sentence presentation in NH subjects and SSD-CI subjects with good and poor performance during unilateral (**A**) and bilateral (**B**) stimulation. Significance of difference for pairwise comparisons: n.s., not significant (*p* > 0.1); **p < 0.01; ***p < 0.001. CI, cochlear implant; NH, normal-hearing; rCBF, relative cerebral blood flow; SSD-CI, single-sided deaf CI subjects; STG, superior temporal gyrus; TR-sentence, time-reversed sentence

To assess the impact of ‘kind of stimulation’ on the left STG, we compared sentence and TR-sentence presentations separately during unilateral and bilateral stimulation. In the NH group, the mean regional rCBF of the left STG was increased during sentence presentation for uni- and bilateral stimulations (Δ = 3.4% ± 1.5%, *t*(138) = 4.1, *p* = 0.001 and Δ = 4.1% ± 2.9%, *t*(138) = 3.4, *p* = 0.01, respectively). Likewise, we found a significant increase of rCBF of the left STG in the sentence compared to the TR-sentence condition in SSD-CI subjects with good performance (Δ = 3.4% ± 1.9%, *t*(138) = 4.8, *p* < 0.001 and Δ = 3.8% ± 3.8%, *t*(138) = 3.7, *p* = 0.004, during uni- and bilateral stimulation, respectively). In contrast, SSD-CI subjects with poor performance showed no significant rCBF increase of the left STG in the sentence compared to the TR-sentence condition irrespective of the side of stimulation (Δ = 2.0% ± 2.9%, *t*(138) = 2.4, *p* = 0.17 and Δ = 2.6% ± 3.4%, *t*(138) = 2.2, *p* = 0.25, during uni- and bilateral stimulation, respectively) (Fig. 6). No quantitative differences in rCBF of the left STG between NH controls and both SSD-CI groups were found, neither for the sentence nor for the TR-sentence condition (*p* > 0.1; but note the small sample size). We found no effects of the repetition on the rCBF in the left STG (*p* > 0.6).

Exploratory voxel-wise analyses in the smaller subgroups (*p* < 0.001 at peak-level, uncorrected; cluster extent of at least 100 voxels) confirmed the aforementioned VOI-based findings, suggesting that the lack of STG activation is not due to an inappropriate VOI (Supplemental Fig. 1).

### Influencing factors

To explore the influence of clinical parameters, we calculated the correlations between available disease-related parameters and magnitudes of activations (regional rCBF difference between stimulation conditions). For this cohort of SSD-CI subjects, we observed no associations between task-specific activations and residual hearing, duration of deafness, CI experience, or age at onset of deafness for neither of the tested regions (Supplemental Fig. 2).

## Discussion

The present study explores the neuronal mechanisms underlying binaural integration in SSD-CI subjects. We applied the OLSA (i.e., German matrix test) and [^15^O]water PET to evaluate blood flow response patterns during word understanding in background noise in SSD-CI subjects with good and poor performance compared to NH controls. Our results show that a CI can provide auditory stimulation to the PAC comparable to a NH ear, but the failure to reduce PAC rCBF asymmetry during bilateral compared to unilateral stimulation suggests altered stimulus processing in SSD-CI subjects with poor performance. This might suggest a persisting dominance of the non-implanted (NH) ear in these subjects. The crucial role of the non-implanted NH ear is further highlighted by lower word understanding and lack of STG activation in SSD-CI subjects with poor performance, irrespective of whether the stimulation was applied to both ears (NH ear and CI ear) or the NH ear only.

### Sentence processing in background noise

One of the most relevant functional deficits caused by SSD affecting daily life is impairment of speech understanding in background noise, which can be improved by CI [14, 15], see Introduction.

To evaluate the performance in word recognition, we used a median split (80%) correct word understanding in PET background noise during bilateral stimulation. SSD-CI subjects with good performance showed better word understanding in PET background noise during bilateral but also during unilateral (NH ear) stimulation. Both NH controls and SSD-CI subjects with poor performance demonstrated high variability in word understanding during unilateral stimulation, most likely caused by individual, not auditory-related factors (e.g., working memory [30]). Word understanding in PET background noise during unilateral stimulation of the non-implanted NH ear did not differ between NH controls and SSD-CI subjects, but SSD-CI subjects with good performance showed **high normal** word understanding in PET background noise during unilateral stimulation, and SSD-CI subjects with poor performance showed **low normal** word understanding in PET background noise during unilateral stimulation. This might imply that the differentiation between good and poor performance can be made before treatment with CI using word understanding in background noise with the non-implanted NH ear.

### Auditory input (‘side of stimulation’)

To investigate the neuronal response of the PAC to auditory stimuli in SSD-CI subjects, we compared unilateral stimulation of the right NH ear and bilateral stimulation of the right NH ear plus left NH ear or left CI. We refrained from unilateral stimulation of the left CI (or left NH ear only), as this is not an everyday life listening situation for SSD-CI subjects, and because the PET background noise could not be blocked sufficiently from the NH ear. Even with earplugs, the PET background noise was only reduced to 60 dB(A) from 74 dB(A), i.e., by 14 dB [23]. In consequence of this insufficient blockage, the PET noise is still present in the contralateral NH ear during unilateral stimulation in NH controls and is therefore a limitation of the present study.

Our results showed increased activation of the contralateral PAC during bilateral stimulation in NH controls and both SSD-CI groups. Cortical activation through stimulation of a CI was also shown for CI users with bilateral deafness [31, 32] and asymmetric hearing loss (AHL) [33]. Unlike Karoui et al. [33], who observed a lower auditory cortical activation in subjects with AHL, we found comparable neuronal activation during acoustic stimulation of a left NH ear in NH controls and electric stimulation of a left CI ear in all SSD-CI subjects. This may be caused by the hearing capacity of the non-implanted ear: in our study, the hearing of the non-implanted ear was undisturbed in SSD-CI subjects, whilst in the study of Karoui et al. [33], the non-implanted ear showed mild to moderate hearing loss.

Besides the “bottom-up” process of auditory activation from the cochlear via the central auditory pathway, “top-down” pathways connect the PAC to the auditory thalamus [34], to the inferior colliculus [35], to the cochlear nucleus, to the superior olivary complex [36, 37], and extend to cochlear receptors and the auditory nerve [38]. These efferent, corticofugal pathways help with signal discrimination and are activated during difficult listening situations [39–41]. Degeneration of these pathways during aging contributes to age-related hearing loss and consequential cognitive decline [42]. In rodents, cortical electrical stimulation of the PAC preserved the cochlear efferent system [42], improved cochlear aging traits [42], showed otoprotective effects after noise overactivation [43], and stabilized hearing loss in rats with age-related hearing loss [44]. Based on these results, we hypothesize that the preserved corticofugal pathways of the PAC contralateral to the NH ear in subjects with SSD might have a protective effect on the auditory nerve of the hearing-impaired ear and can prevent cortical activation decay after deafness [45]. This ‘protective efferent effect’ would be less pronounced in subjects with impaired hearing in the non-implanted ear [33] or bilateral deafness [31, 32], further explaining the differences between the present results and those of Karoui et al. [33].

It is established that the auditory pathway shows contralateral lateralization because of predominantly crossed projections [46]. Unilateral hearing loss disturbs this contralateral lateralization, resulting in a more bilateral activation response to unilateral stimulation of the remaining intact ear [47–49]. Treatment with a CI can (partly) reverse the pathological bilateral neural response and restore lateralization in AHL [33, 50]. In our study, we detected contralateral lateralization to the left PAC during unilateral stimulation of the right NH ear in NH controls and SSD-CI subjects with poor and good performance. As would be expected, the AI-PAC was reduced during bilateral stimulation in NH controls and SSD-CI subjects with good performance. However, this drop in AI-PAC was not observed in SSD-CI subjects with poor performance. Interestingly, the AI-PAC was skewed to the left during bilateral stimulation in all groups; most likely as a neural correlate for the right-ear advantage seen in subjects with postlingual SSD [51] and NH controls. This confirms our hypothesis that SSD-CI subjects with poorer performance show altered activation patterns of PAC. However not the activation of the right PAC (contralateral to the left CI) differed, as the mere quantity was comparable to NH controls and SSD-CI subjects with good performance, but the concurrent stimulation of the left PAC resulted in a constantly high AI-PAC during bilateral stimulation.

### Sentence processing (‘kind of stimulation’)

Beyond sound recognition, our study explored the neuronal activation during the processing of syntactically correct sentences and TR-sentences. We applied the Oldenburg sentence test, which is the matrix test commonly used in clinical routine and, therefore, well known by all participating SSD-CI subjects. All subjects included in the present study were trained on the material of the OLSA the day before the PET measurement and were made aware that the sentences were grammatically correct but void of meaning.

For NH controls and SSD-CI subjects with good performance, we observed an activation of the left STG during sentence compared to TR-sentence presentation during unilateral and bilateral presentations. In contrast, SSD-CI subjects with poor performance showed no difference in activation of the left STG during sentence compared to TR-sentence presentation, neither during unilateral NH ear nor during bilateral stimulation.

The activation cluster of the left STG during sentence presentation in our study overlapped with the region T1_4 defined by Labache et al. [52]. This region is described to distinguish between sentences that were understood and not understood [53–55], which is in line with the stimulation presented in our study. In contrast, the region STS4 is responsible for the interpretation of sentences, and therefore, we expect from the sentence material presented in our study (grammatically correct but known meaningless sentences) that the STS4 would not be involved. Our findings are in good agreement with the literature, suggesting that the activation of the left STG is a reflection of sentence recognition in PET background noise.

This partially confirms our second hypothesis: SSD-CI subjects with poor performance show less activation of the left STG during word understanding in background noise than NH controls, and interestingly, SSD-CI subjects with good performance. Taking the literature on the role of the left STG into consideration, this difference in neuronal activation might be a reflection of successful word understanding and merely an objectification of the subjective hearing test. It would be interesting to investigate in future studies at which time point the differences in activation of STG occur – before unilateral deafness onset, before treatment with CI, and/or after treatment with CI.

### Clinical implications

Based on the literature in animals and humans, we expected that the age of deafness onset as well as the duration of deafness would influence neuronal activation of the auditory pathway. In postlingual deafness, older age at the onset of deafness is associated with longer preservation of tonotopy in the central auditory pathway because plasticity levels are lower in older individuals [56–60]. Vice versa, younger age at onset of deafness duration and presumed higher levels of brain plasticity lead to quicker loss of tonotopy in the central auditory pathway [61–63]. This might explain why older CI users display similar or better outcomes with CI than younger CI users [64–68]. In addition, the duration of deafness impacts neuronal activation, as literature shows that the shorter the duration of deafness, the more preserved the tonotopy of the central auditory pathway [49]. Importantly, the interaction of duration of deafness and age at onset of deafness has to be taken into account. Nevertheless, in the present study, these disease-related factors commonly used in the clinical setting had no significant influence on the magnitudes of activations (regional rCBF difference between stimulation conditions) in the PAC or STG. This is most likely because SSD-CI subjects with good and poor performance did not significantly differ in these factors.

However, SSD-CI subjects with good and poor performance showed significant differences in word understanding and neuronal activation in background noise. Virtually all SSD-CI subjects improved in sentence recognition in PET background noise during bilateral stimulation. However, even though word understanding during unilateral stimulation for both SSD-CI groups did not differ from NH controls, subjects with poor performance showed significantly poorer word understanding during unilateral stimulation than those with good performance. This difference between **high and low normal** word understanding in background noise during unilateral NH ear stimulation could be used to improve patient information before operation, identify suitable CI candidates, and personalize rehabilitation after CI therapy.

On the level of the PAC, we saw comparable AI-PAC in all groups during unilateral stimulation, which did not allow for discrimination between the present SSD-CI subjects with poor or good performance. This is at variance with an earlier study of our group, in which the asymmetry of neural activity of the PAC (at ambient noise) predicted CI success in subjects with AHL who *later* underwent CI treatment [25]. Thus, it is tempting to speculate that differences in PAC findings in the present and the earlier study are due to neuronal changes induced by longstanding CI treatment in the present cohort as opposed to the CI-naive subjects in the earlier study.

On the level of the left STG, the lack of activation in SSD-CI subjects with poor performance, in contrast to NH controls and SSD-CI subjects with good performance, may also possess predictive value, although STG activation may simply reflect word understanding in background noise similar to a conventional hearing test.

Taken together, a prospective study in CI-naive SSD subjects is needed to explore and validate whether high vs. low normal word understanding in the background during unilateral stimulation of the NH ear predicts performance with CI. Including [^15^O]water PET in such studies would offer the very attractive opportunity to gain further insights by additionally investigating the possible predictive value of STG activation and AI-PAC during stimulation of the NH ear.

## Conclusion

This study underlines the usefulness of [^15^O]water PET to explore regional neuronal mechanisms underlying CI performance: Our results suggest that SSD-CI subjects with poor performance are characterized by a low normal word understanding when using the NH ear only (in line with the lack of STG activation) and compromised binaural integration (as witnessed by the lack of AI-PAC decline upon bilateral stimulation), although the CI provides quantitatively comparable input to the contralateral PAC in both SSD-CI groups. Further studies are needed to explore whether word understanding in noise with the NH ear may satisfy the medical need of a convenient and valid predictor of CI outcome in SSD.

## Supplementary Information

Below is the link to the electronic supplementary material.

Supplementary Material 1 (PNG 5.41 MB)

Supplementary Material 2 (PNG 2.11 MB)

## Data Availability

The datasets generated during and/or analysed during the current study are available from the corresponding author on reasonable request.
