# Supplementary figures and images for: Neuronal mechanisms underlying binaural performance with cochlear implant in single-sided deafness: a [15O]water PET study
Source: Eur J Nucl Med Mol Imaging. 2025 Nov 15;53(4):2751–63. doi: 10.1007/s00259-025-07639-8 (PMC12920324; doi:10.1007/s00259-025-07639-8)

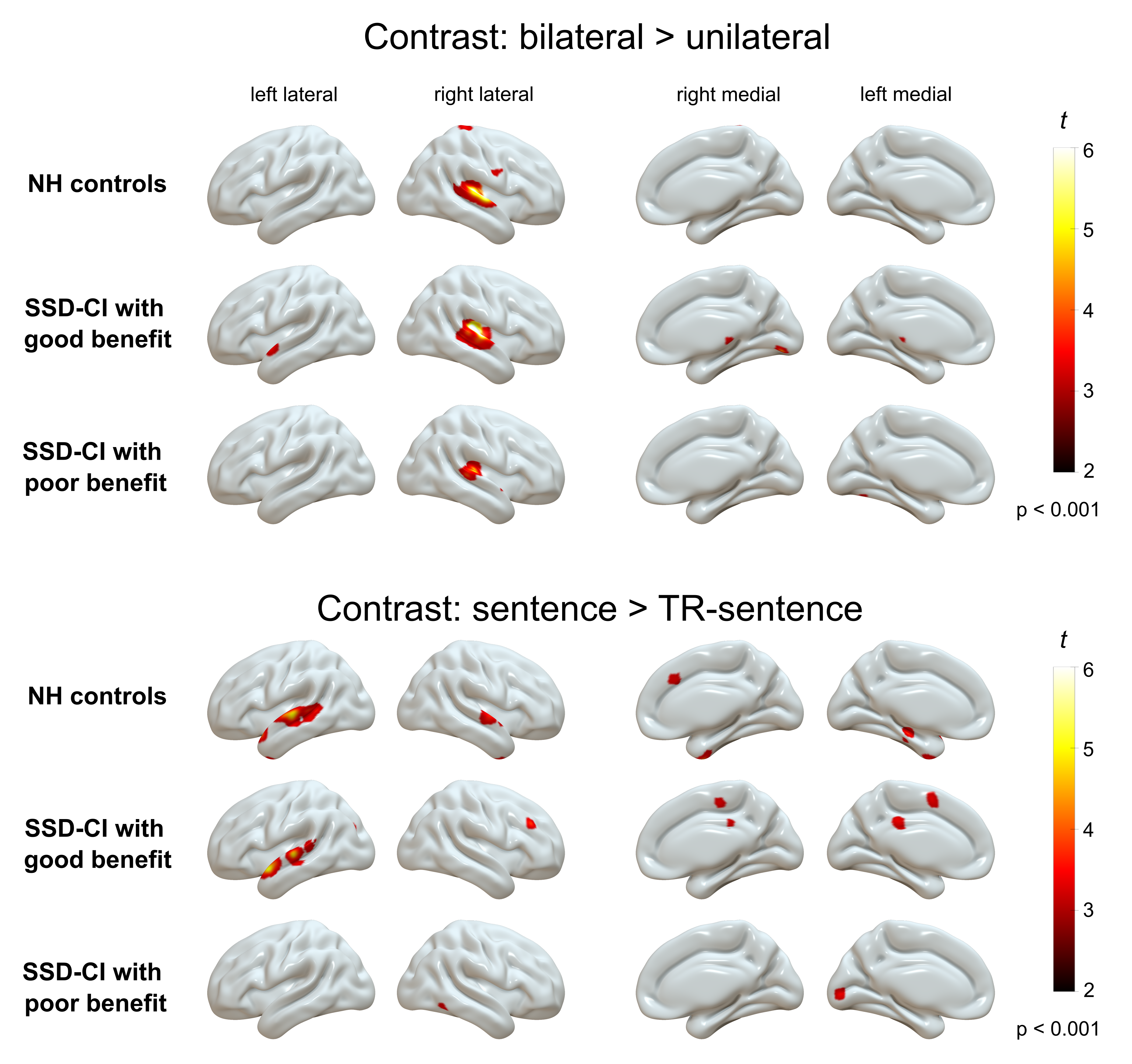

Supplement: Supplementary file 1 — Supplementary Material 1 (PNG 5.41 MB) [file 259_2025_7639_MOESM1_ESM.png]

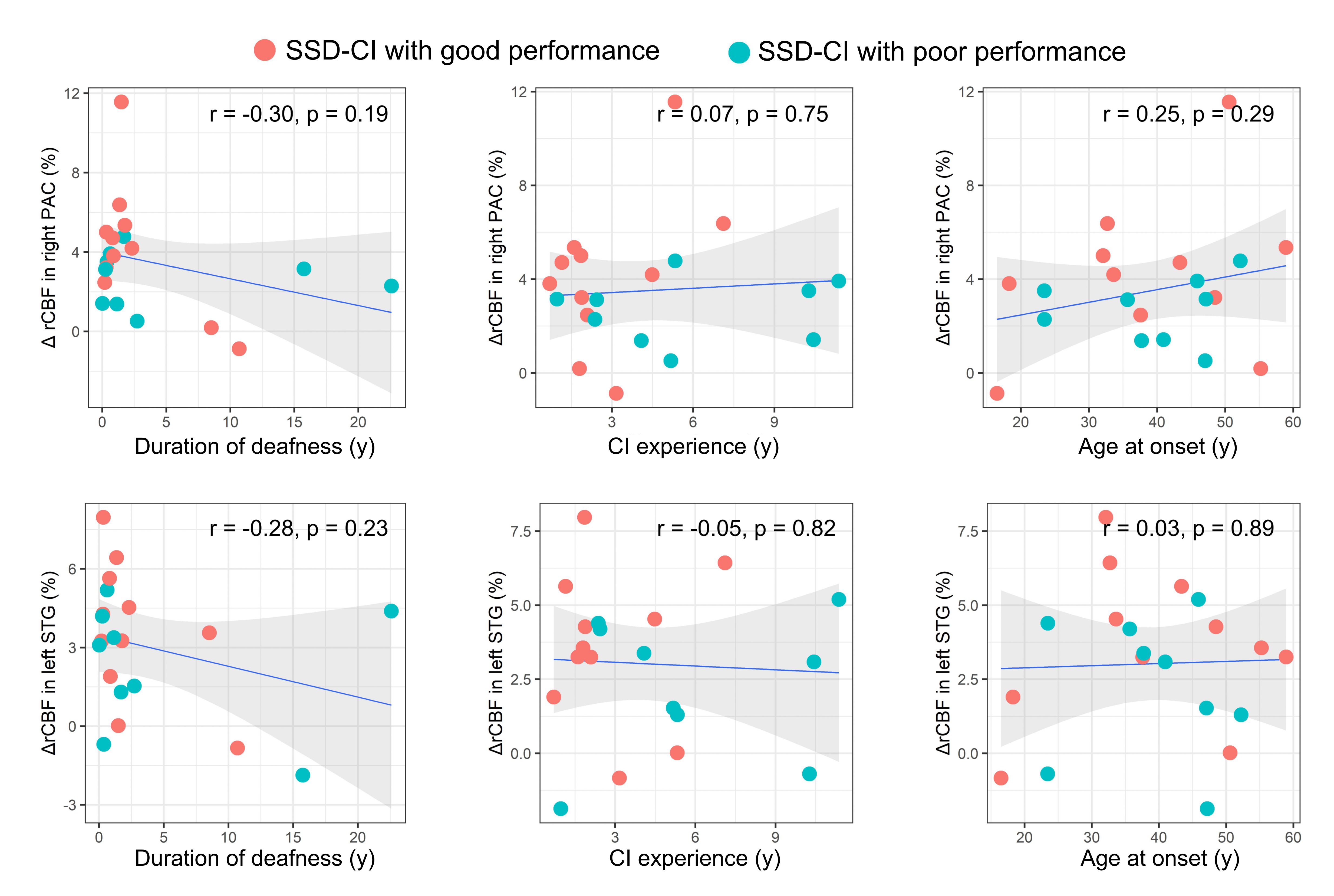

Supplement: Supplementary file 2 — Supplementary Material 2 (PNG 2.11 MB) [file 259_2025_7639_MOESM2_ESM.png]
